# Supplementary material for: Deformation Driven Suction Cups: A Mechanics‐Based Approach to Wearable Electronics
Source: Adv Sci (Weinh). 2026 Feb 10;13(20):e20417. doi: 10.1002/advs.202520417 (PMC13067814; doi:10.1002/advs.202520417)
Supplement: Supplementary file 1 — Supporting File 1: advs74135‐sup‐0001‐SuppMat.pdf. [file ADVS-13-e20417-s002.pdf]

SI for Deformation Driven Suction Cups: A Mechanics-Based Approach to Wearable Electronics

Seola Lee[a,b], Andrew Akerson[a], Roham Pardakhtim[a], Ehsan Hajiesmaili[a], Kevin Rhodes[a], Zidong Li[a], Andrew Stanley[a], Amirhossein Amini[a], Daniele Piazza[a], Chiara Daraio[a,b], and Tianshu Liu[a,*]

[a]Reality Labs Research, Meta Platforms, Inc., Redmond, WA 98052, USA
[b]Division of Engineering and Applied Science, California Institute of Technology, Pasadena, CA 91125, USA
[*]Corresponding author: Tianshu Liu, Tianshu.liu@meta.com

1 Suction cup fabrication

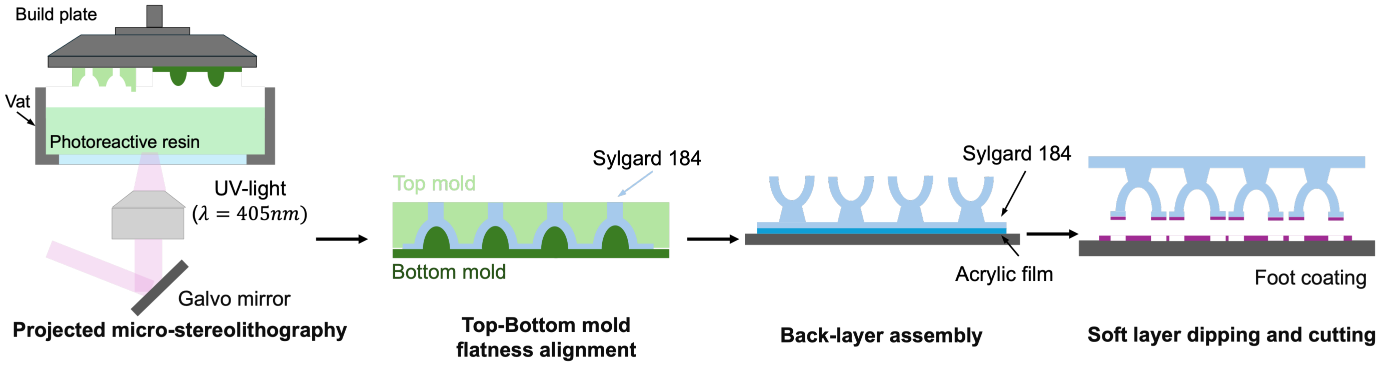

Figure S1: Suction cup fabrication processes.

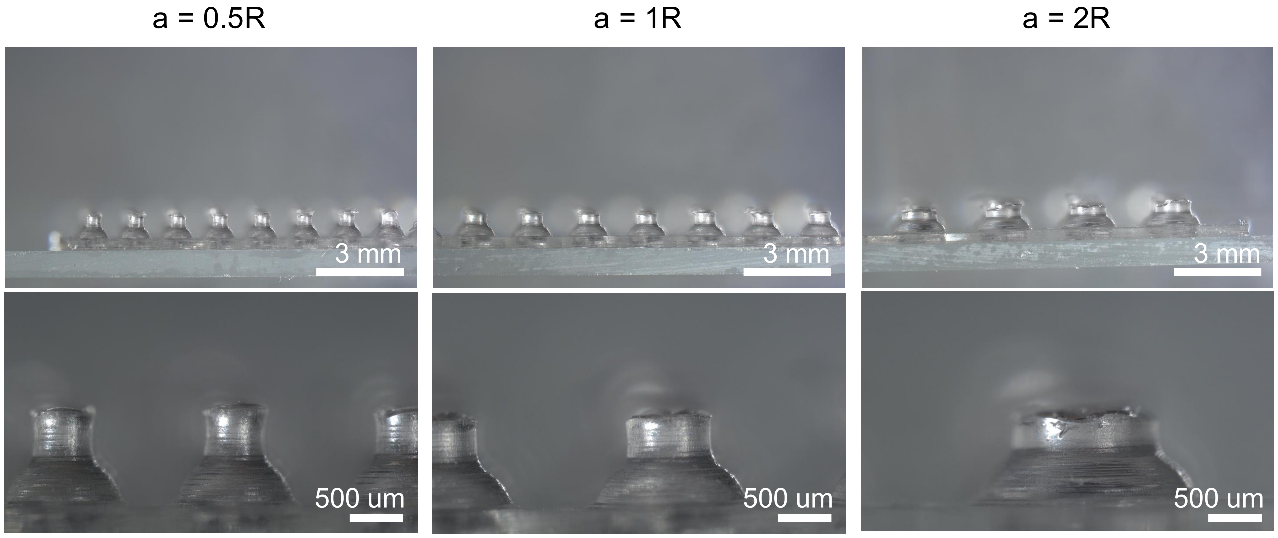

Figure S2: Optical images and measurements for main bodies of the cups with $R = 300\mu m$ cups with varied a

2 Normal pull-off measurements

2.1 Testing Set-up

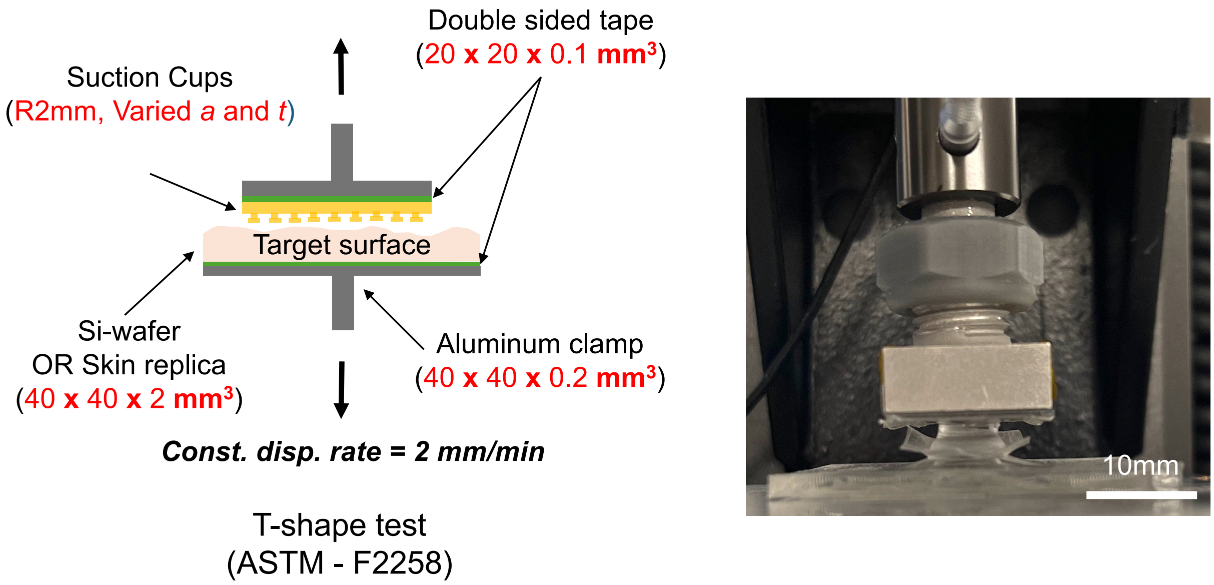

Figure S3: Pull-off strength measurement set-up

2.2 Geometrical Design Effects on Adhesion Strength

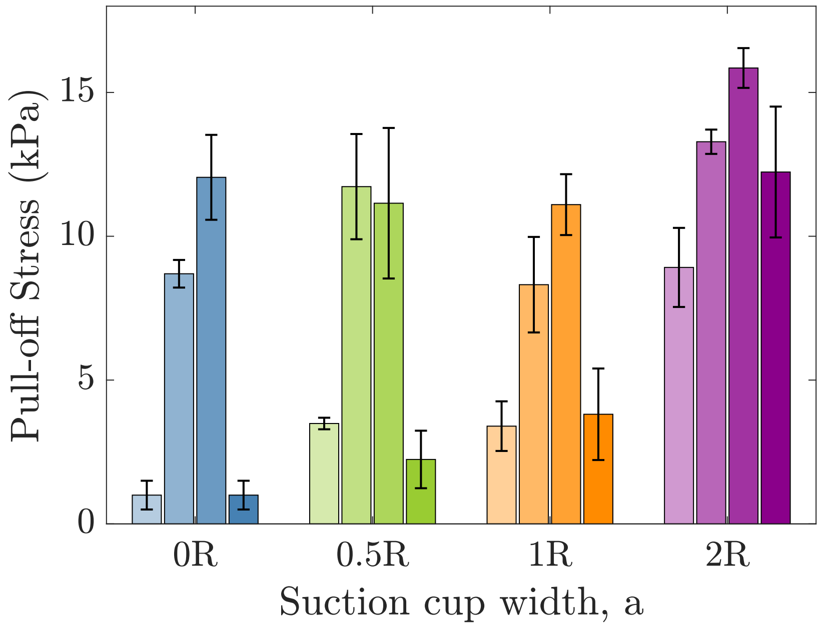

Figure S4: Histogram of pull-off strength for suction cups with varying roof width (a) and shell thickness (t) on a silicon wafer. Bar color indicates thickness: from lightest to darkest, $t = 0.1R$, $0.2R$,$0.3R$, $0.4R$ for $R = 2mm$.

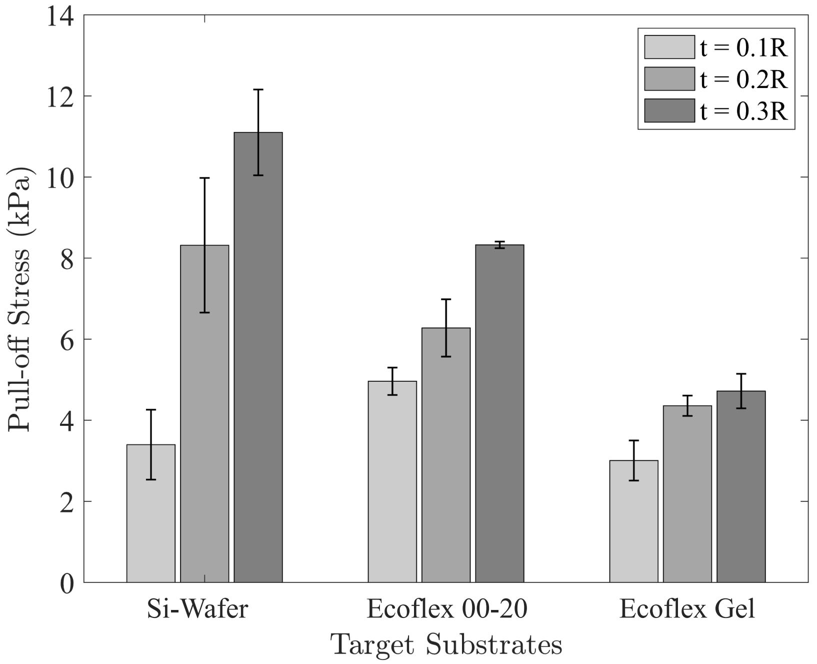

Figure S5: Histogram of pull-off strength for suction cups with varying cup thickness (t) on different target stiffness. Bar color indicates thickness: from lightest to darkest, $t = 0.1R$, $0.2R$,$0.3R$ for $a = R$ and $R = 2mm$.

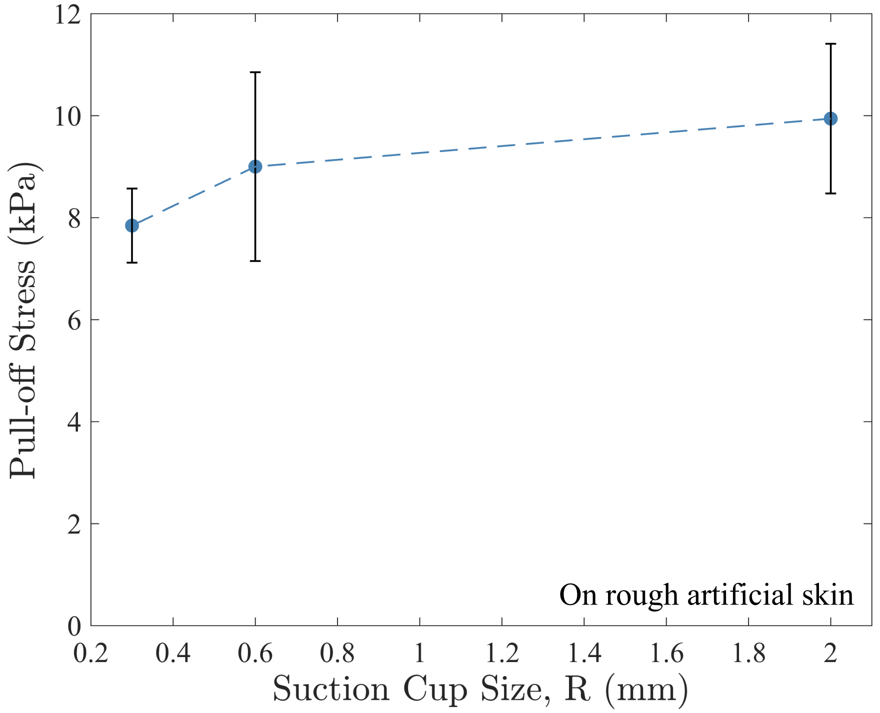

Figure S6: Suction cup arrays with varying cup radii (R) and fixed geometry ratios ($a = R$,$t = 0.3R$) tested on rough artificial skin.

2.3 Repeatability of Adhesion Strength

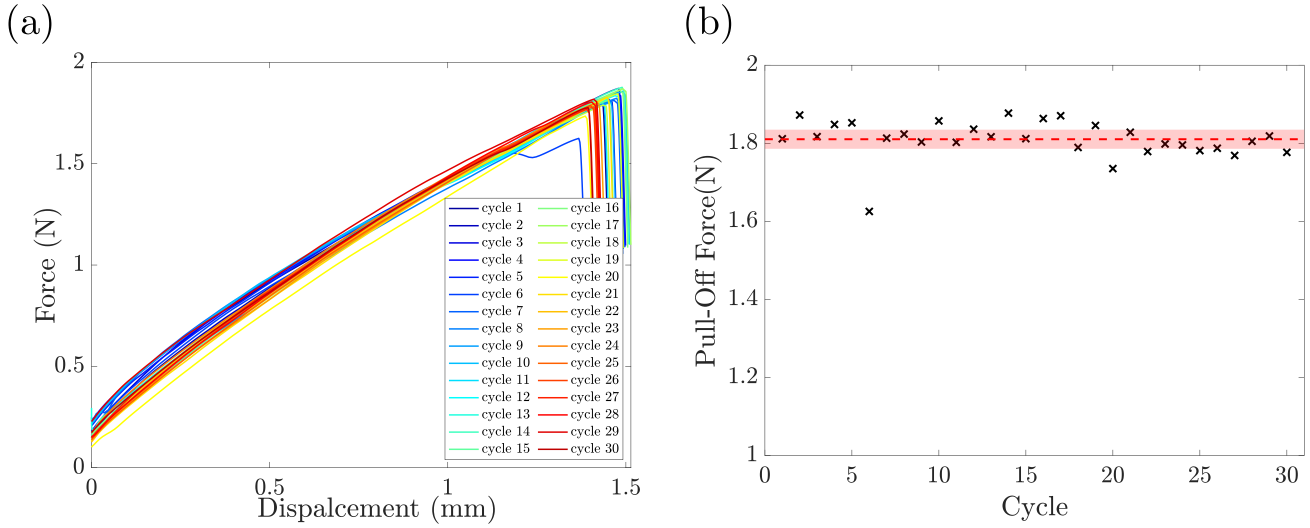

Figure S7: Repeatability of suction cup ($a = R = 2$ mm, $t = 0.3R$) performance tested on rough artificial skin. (a) Force–displacement curves for each test. (b) Pull-off force for each test (black ×'s), with mean (red dashed line), and 99% confidence interval (shaded red region).

2.4 Aging Effects on Adhesion Strength

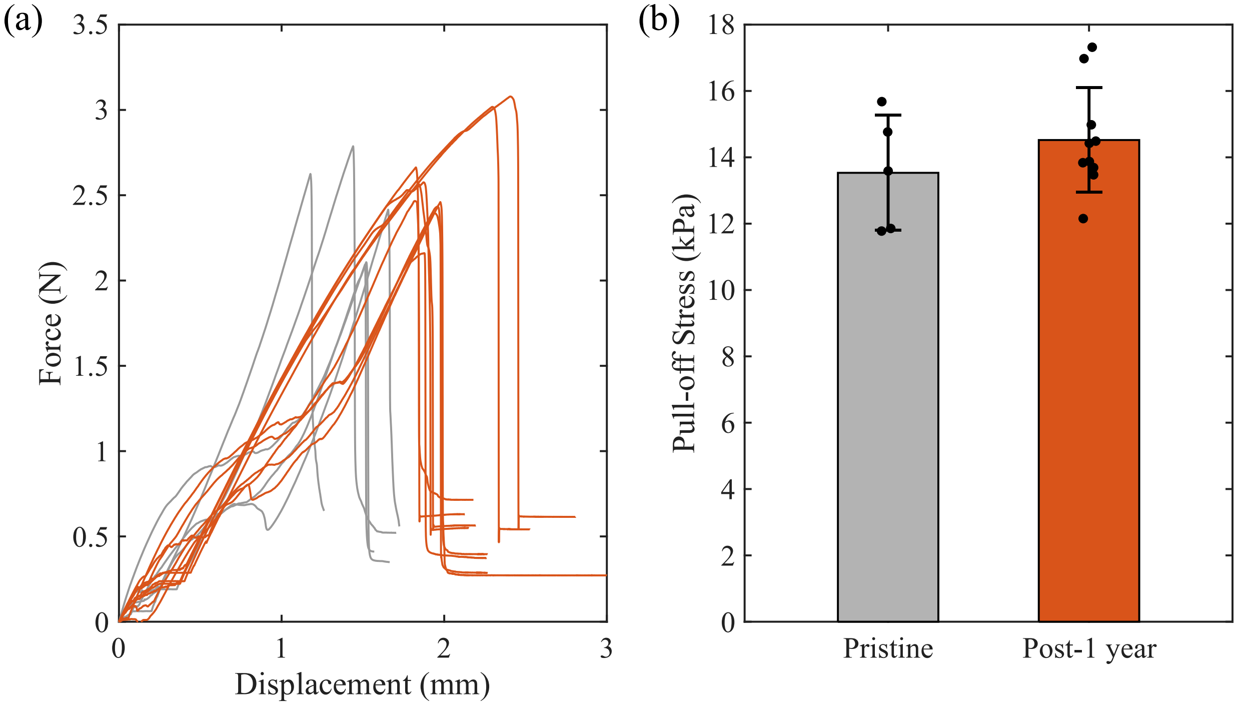

Figure S8: Aging effects of suction cup ($a = R = 2$ mm, $t = 0.3R$) performance tested on rough artificial skin. (a) Force–displacement curves for pristine (gray) and post-1-year aged (orange) samples. Each curve corresponds to one adhesion cycle. (b) Bar plot of average pull-off stress for pristine and post–1-year samples, calculated from the peak detachment force of each adhesion cycle.

The long-term stability of our adhesive system is primarily governed by the mechanical stability of the structural elastomers, as adhesion arises from a geometry-mediated suction mechanism. We therefore deliberately selected commercial silicone elastomers (PDMS and Ecoflex) that are widely used in wearable and soft robotic systems due to their well-established robustness under repeated deformation. Previous aging studies on silicone elastomers report only modest

changes in elastic modulus, strength, and extensibility ($< \pm 6\%$) after natural aging in air for several weeks, with similarly limited property changes ($< \pm 10\%$) even under prolonged underwater exposure [**zhang˙PDMS˙agingeffects˙2025**]. In addition, cyclic mechanical studies have demonstrated stable stress–strain behavior of silicone elastomers, including Sylgard 184, over extended uniaxial cycling (up to $\sim 2.2 \times 10^5$ cycles) without significant degradation in mechanical response [**Bernardi˙cyclic˙elastomer˙2017117**]. Consistent with this material-level stability, silicone-based epidermal and skin-conformal electronic platforms have repeatedly demonstrated durability under repeated mounting, handling, and daily-use deformations in prior wearable device studies [**Kim˙SkinConformal˙2020**, **Yeo˙Epidermal˙electronics˙2013**]. Because the suction adhesive relies on structural compliance rather than interfacial chemistry, the relatively stable mechanical properties of the elastomers enable repeatable adhesion over extended reuse, as also reported in prior suction- and microstructure-based adhesive systems that demonstrate robust wet/dry adhesion under repeated attachment and detachment [**baik˙wet-tolerant˙2017**]. In practical use, adhesion degradation is expected to be dominated by surface fouling (e.g., dust, skin oils, perspiration) rather than irreversible material damage; such effects are commonly mitigated by simple rinsing or cleaning protocols, consistent with the operating principles of reusable micro-suction interfaces. To further support this point, we re-measured the adhesion strength of materials fabricated approximately one year prior and compared to freshly prepared samples. Fig. S8 shows that suction cups tested after approximately one year exhibit force–displacement responses and pull-off stresses comparable to those of freshly prepared samples when tested on rough artificial skin. The overall loading stiffness, peak detachment force, and abrupt release behavior remain unchanged (Fig. S8a), indicating preservation of the suction-dominated adhesion mechanism over time. The averaged pull-off stress shows no measurable degradation after aging, with a slightly higher mean value observed for post-1-year samples (Fig. S8b). This modest increase is not interpreted as intrinsic strengthening, but likely reflects secondary factors such as small variations in ambient humidity, substrate compliance, or sample-to-sample geometric conformity, all of which can influence rim sealing in suction-based adhesion. Overall, these results demonstrate that long-term storage under ambient conditions does not compromise adhesion performance, supporting the mechanical durability and reuse reliability of the suction adhesive system.

2.5 Hydration Effects on Adhesion Strength

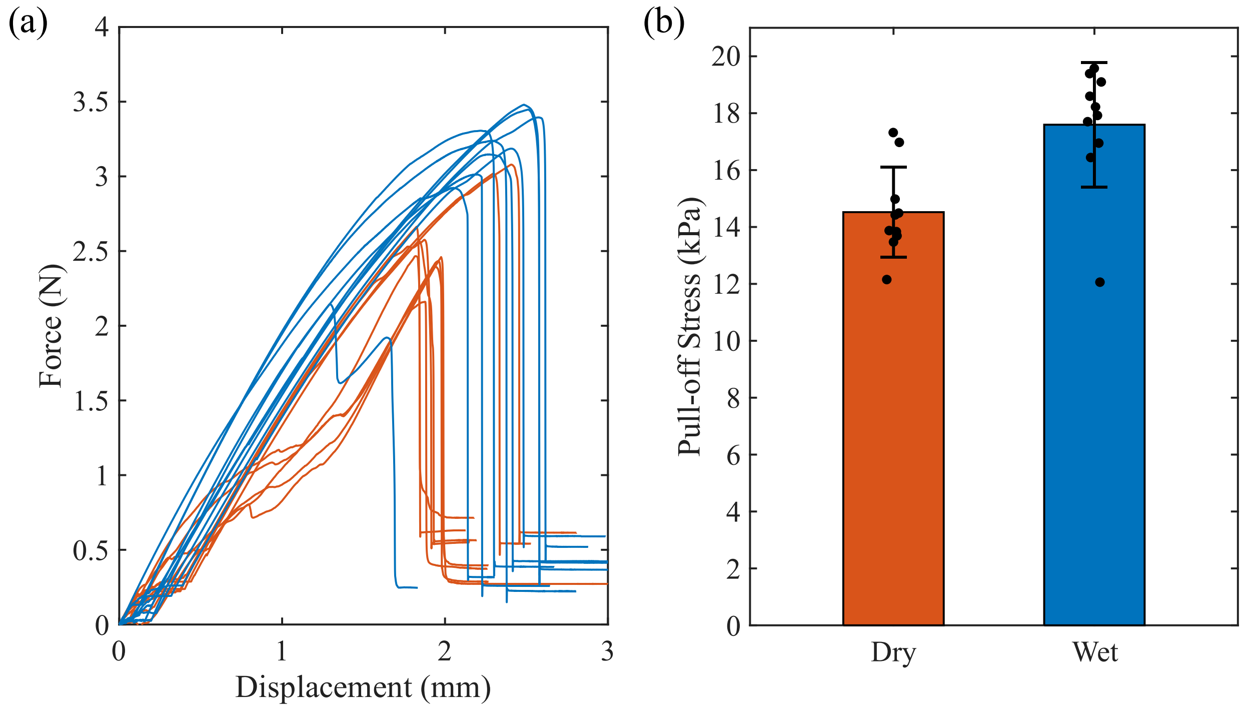

Figure S9: Hydration effects on suction-cup adhesion ($a = R = 2$ mm, $t = 0.3R$) on rough artificial skin. (a) Force–displacement responses measured on dry (orange) and wetted (blue) substrates; each curve represents an individual adhesion cycle. (b) Bar plot summarizing the average pull-off stress for dry and wet conditions, calculated from the peak detachment force of each adhesion cycle.

In wearable applications, the skin–device interface is frequently exposed to perspiration, resulting in micro-droplets or a thin liquid layer at the contact surface. To examine the influence of such hydrated conditions on suction-based adhesion, we measured adhesion strength on wetted substrates (Fig. S9). Under wet conditions, normal pull-off force is expected to be more stable or even higher due to improved sealing at the rim of the suction cup. Consistent with this expectation,

that the adhesion strength increases from 14.52 ± 1.58 kPa under dry conditions to 17.60 ± 2.19 kPa when the substrate is wetted. Mechanistically, a thin fluid film can infiltrate micro-asperities and increase the hydrodynamic resistance associated with flow through rim. Both effects can stabilize the pressure differential across the cup cavity, thereby stabilize or improve the normal adhesion strength. In contrast, shear resistance may be reduced under sweating or high-humidity condition, as fluid transitions part of the interface toward a lubrication-dominated regime. This may make tangential slip becomes easier, even if suction pressure and normal adhesion remain high as demonstrated in suction systems operating under wet and fully underwater conditions [**yue˙bioinspired˙2024**]. Systematic studies that independently control perspiration loading and dynamic shear on skin-like substrates would be beneficial to map the operation limits for our suction cups.

3 Suction cup modeling

We model the suction cup through a quasi-static, axisymmetric formulation. We consider two distinct loading states. In the first, the un-deformed cup structure is loaded while allowing air to escape the enclosed region. In the second phase, air may not enter or leave the cavity. Through the elastic recovery of the unloaded structure, a partial vacuum is formed in the enclosed region, as shown Figure S10. We first model the loading stage through large-deformation kinematics while allowing

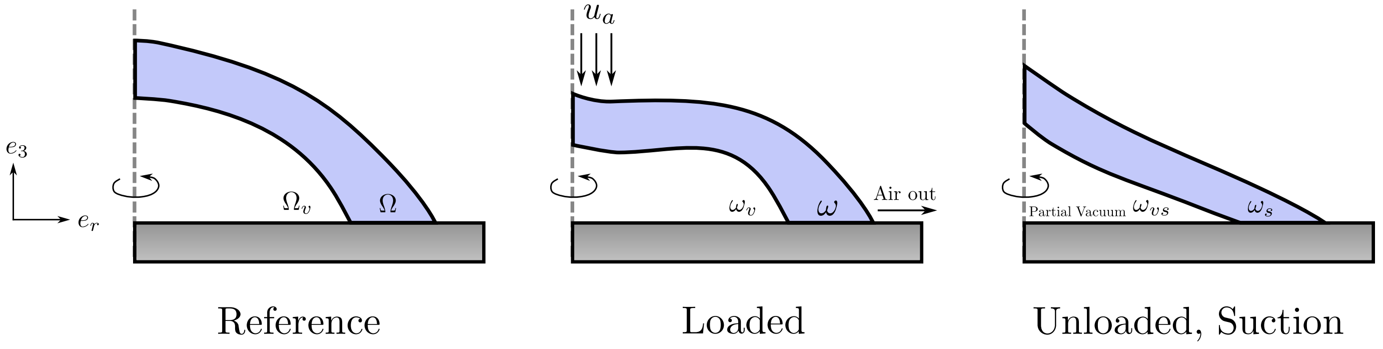

Figure S10: Diagram of the axisymmetric suction cut in the reference, loaded, and unloaded suction states.

free variation of the enclosed volume. Then, we model the unloading vacuum stage by coupling the cavity volume with an internal pressure evolution through an equation of state.

3.1 Loading Stage

The formulation in the loading stage is a standard finite-elasticity problem. We consider an axisymmetric structure occupying Ω in the reference configuration that undergoes an applied displacement u_a on its top boundary $\partial_u\Omega$. We consider finite-deformation kinematics with an internal energy density function W^{cup}. The free energy of the structure is the elastic energy of the cup,

$$\mathcal{E}^L = \mathcal{E}^{el} := 2\pi \int_\Omega W^{cup}(F) R \, d\Omega \tag{1}$$

where $F(\nabla u, u_r)$ is the deformation gradient tensor, dependent on the displacement gradient ∇u and the radial displacement u_r in the axisymmetric setting. For now, we leave the form of the energy function W^{cup} general, and choose a particular constitutive law in the following sections. Taking variations gives the weak-form of equilibrium

$$0 = 2\pi \int_\Omega R \frac{\partial W^{cup}}{\partial F} \cdot \left(\frac{\partial F}{\partial \nabla u} \cdot \nabla \delta u + \frac{\partial F}{\partial u_r} \delta u_r \right) \, d\Omega \qquad \text{for all } \delta u \in \mathcal{U}_0, \tag{2}$$

where $\mathcal{U}_0$ is the space of kinematically admissible displacement variations

$$\mathcal{U}_0 := \left\{ u \in H^1(\Omega), \; u = 0 \text{ on } \partial_u\Omega \right\}, \tag{3}$$

where $H^1(\Omega)$ is the standard vector-valued Hilbert space.

3.2 Suction Stage

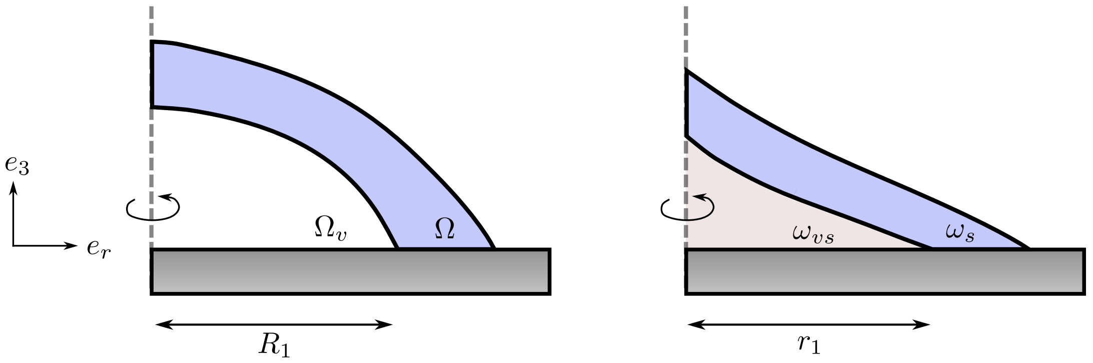

Figure S11: Diagram showing the reference and unloaded suction regions.

In the suction stage, careful consideration must be made when handling the pressure and volume in the enclosed region. We consider the isothermal work done by the structure onto the gas in the enclosed region as

$$\mathcal{E}^{gas} := -\int_{V_0}^{V} (p - p_0)\ dV, \tag{4}$$

where p is the pressure in the enclosed region, p_0 is the ambient pressure, V_0 is the enclosed volume at the initiation of the suction stage, and V is the current volume of the enclosed region. Assuming an ideal gas,

$$pV = p_0 V_0. \tag{5}$$

Then,

$$\mathcal{E}^{gas} = -\int_{V_0}^{V} \left(\frac{p_0 V_0}{V} - p_0\right)\ dV = -p_0 V_0 \log\frac{V}{V_0} + p_0(V - V_0). \tag{6}$$

The total energy of the system in the suction phase is the sum of the elastic energy of the cup and the work on the gas,

$$\mathcal{E}^S = \mathcal{E}^{el} + \mathcal{E}^{gas} = 2\pi \int_\Omega W^{cup}(F) R\ d\Omega - p_0 V_0 \log\frac{V}{V_0} + p_0(V - V_0). \tag{7}$$

where V is the deformed volume of the enclosed region ω_{vs},

$$V := \int_{\omega_{vs}} 2\pi r\ d\omega \tag{8}$$

in the cylindrical coordinate system assuming axisymmetry. We pull this back to the reference configuration as

$$V = \int_{\Omega_v} \det(F_{2D}) 2\pi (R + u_r)\ d\Omega, \tag{9}$$

where F_{2D} is the 2D deformation gradient for the mapping from Ω_v to ω_{vs}. We may insert this relation into (7) and take variations to obtain the weak form of equilibrium. However, this system is highly non-linear, with the equation of state being directly coupled to the expression for the enclosed volume. This system is difficult to solve, and may not converge with a standard Newton-Raphson scheme. To mitigate this, we reintroduce the pressure p, which acts as a Lagrange multiplier constraining an auxiliary variable $\bar{V}$ to the enclosed volume. This changes the system energy to

$$\mathcal{E}^S = 2\pi \int_\Omega W^{cup}(F) R\ d\Omega - p_0 V_0 \log\frac{\bar{V}}{V_0} + p_0(V - V_0) - p(V - \bar{V}). \tag{10}$$

Then, rearranging terms gives

$$\mathcal{E}^S = 2\pi \int_\Omega W^{cup}(F) R\ d\Omega - p_0 V_0 \log\frac{\bar{V}}{V_0} - (p - p_0)(V - \bar{V}) - p_0(V_0 - \bar{V}). \tag{11}$$

Substituting (9) in for V and taking variations with u, p, and $\bar{V}$ gives the equilibrium relations

$$\begin{aligned} 0 &= 2\pi \int_\Omega R \frac{\partial W^{cup}}{\partial F} \cdot \left(\frac{\partial F}{\partial \nabla u} \cdot \nabla \delta u + \frac{\partial F}{\partial u_r} \delta u_r\right)\ d\Omega \\ &\quad - (p - p_0) \int_{\Omega_v} \det(F_{2D}) 2\pi \left(\delta u_r + (R + u_r) F_{2D}^{-T} \cdot \nabla \delta u\right)\ d\Omega_v, \qquad \text{for all } \delta u \in \mathcal{U}_0, \\ 0 &= \bar{V} - \int_{\Omega_v} \det(F_{2D}) 2\pi (R + u_r)\ d\Omega \\ 0 &= p - \frac{p_0 V_0}{\bar{V}}. \end{aligned} \tag{12}$$

The first equation is the balance of linear momentum, the second relation constrains the auxilliary volume $\bar{V}$ to the enclosed volume, and the last equation is the ideal gas law equation-of-state. Thus, the above is a non-linear set of equations for the displacement field, the pressure, and the volume in the enclosed region.

3.3 Pull-off Condition

In the suction stage, the top portion is incrementally pulled upwards until the pull-off condition is met. This occurs when the stretch in the cup overcomes the vacuum of the enclosed gas. In this case, it is when the net contact force between the cup and the substrate becomes non-compressive, that is, when tension must be applied to the bottom of the cup to maintain contact. We define this as when the net reaction force on the bottom of the cup is zero,

$$0 = F_R = 2\pi \int_{\partial_b \Omega} e_3 \cdot \frac{\partial W^{cup}}{\partial F} e_3 R\ dR, \tag{13}$$

where $\partial_b \Omega$ is the boundary on the bottom of the cup.

3.4 Soft Substrates and Footing Layers

For both the soft substrate and footing layer, we consider additional layers of soft, hyper-elastic materials. That is, we consider the elastic energy as

$$\mathcal{E}^{el} := 2\pi \left[\int_{\Omega} W^{cup}(F) R \, d\Omega + \int_{\Omega_f} W^{foot}(F) R \, d\Omega_f + \int_{\Omega_{sub}} W^{sub}(F) R \, d\Omega_{sub} \right], \tag{14}$$

where W^{foot} and W^{sub} are the energy density functions of the footing layer and the substrate. Then, the equilibrium condition is derived in the same manner as detailed in the previous section.

3.5 Numerics and Solution Strategy

The energy in (7) does not have any elastic contributions in the enclosed region Ω_v. This leads to an ill-posed problem, as an infinite number of deformations in this region may give the same enclosed volume V. A standard method to overcome this is to introduce a very soft elastic solid in the enclosed region. We consider an additional elastic energy in the enclosed region

$$\mathcal{E}^{enc} = 2\pi \int_{\Omega_v} W^{enc}(F) R \, d\Omega, \tag{15}$$

with W^{enc} being much softer than W^{cup}. For our computations, we consider a compressible Neo-Hookean constitutive law with a Poisson's ratio $\nu = 0.495$ for the cup and substrate. The solid in the vacuum regions is also considered near-incompressible with $\nu_v = 0.495$ to prevent excessive deformation. With μ being the shear modulus of the cup, we consider a shear modulus in the vacuum region of $\mu_v = 10^{-5}\mu$, which is found to have a negligible effect on the internal cup pressure.

To solve the equilibrium relations (12), we consider a finite element formulation with standard $Q = 1$ quadrilateral elements. We mesh the entirety of the domain, including the cup, the vacuum region, and the substrate. We solve the system of equations with fully coupled Newton-Raphson iterations. That is, considering the displacement field, pressure, and volume as the unknowns to solve the system of equilibrium relations.

4 Preparation and surface roughness of artificial skin

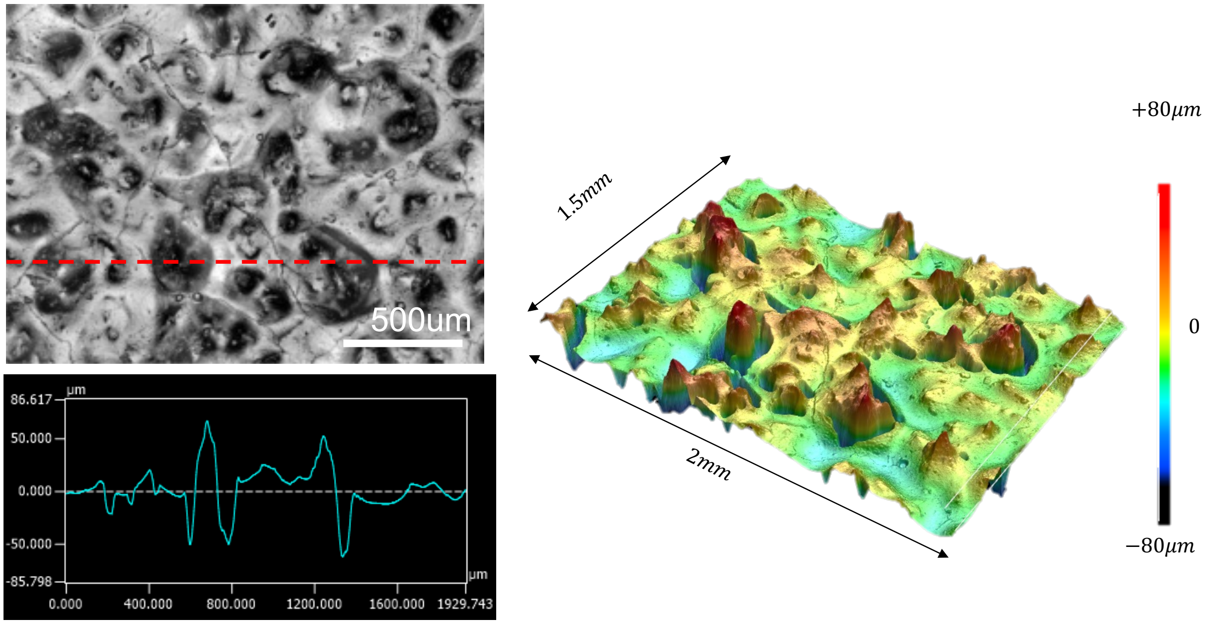

Figure S12: Confocal microscope scan of artificial skin (Vitro Skin) for roughness mapping

5 Gelsight measurement for substrate deformation under suction

The setup consists of a displacement controlled linear actuator positioned on the left side of the system, responsible for providing precise transitional motion. Attached to the actuator, is a high precision Nano 17 force sensor which enables the real time monitoring of the compressive load applied during the testing. Connected to the force sensor is a flat surface compressor which interfaces with a suction cup sample. This suction cup is mounted directly onto the fine tactile sensing gel substrate part of the Gelsight to capture high resolution scans of the deformation. During the operation, the linear actuator

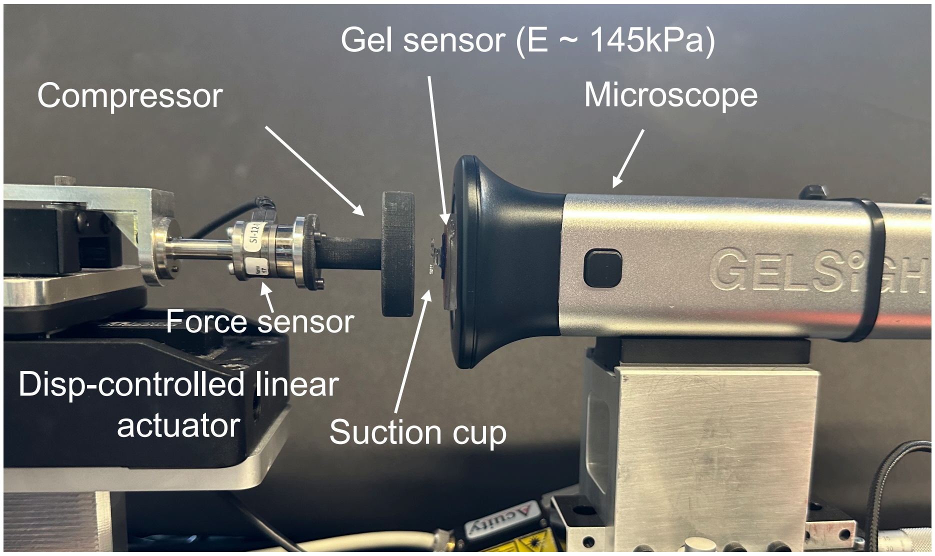

Figure S13: Gelsight measurement set-up

advances the compressor toward the suction cup mounted on the Gelsight sensor. As the compressor contacts the suction cup, the force sensor continuously measures the applied normal force. Once force reaches a predetermined threshold value, the actuator stops the motion and retracts rapidly to allow the cup deformation recovery and vacuum generation within the cup chamber. At this point, Gelsight captures a detailed scan of the substrate deformation, enabling high-fidelity analysis of the mechanical interactions between the substrate and the suction cup sample.

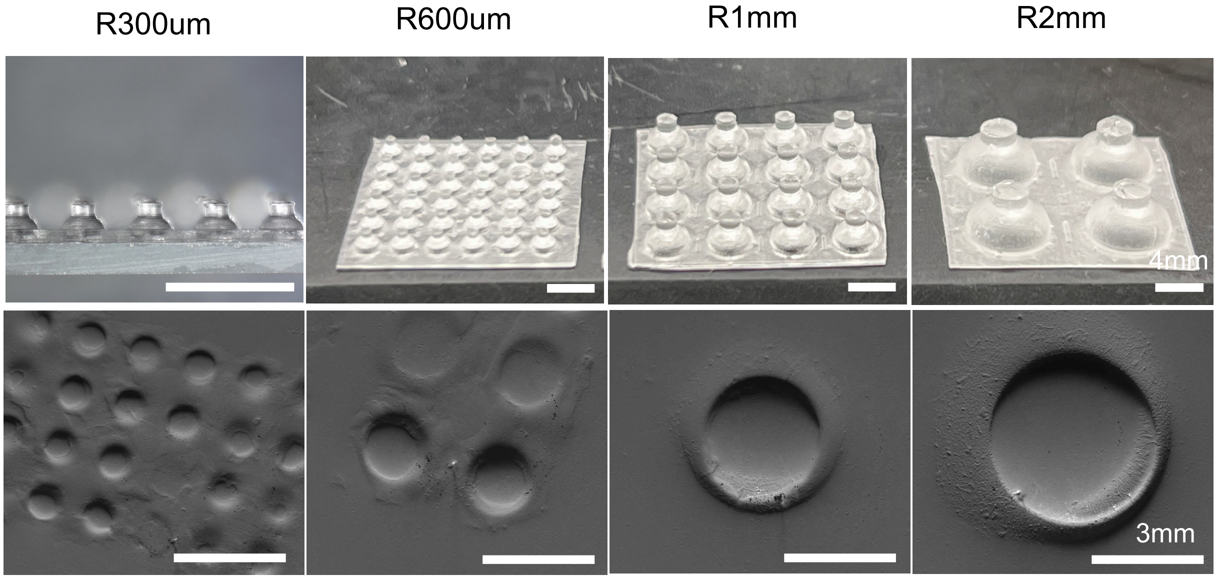

Figure S14: GelSight measurement of substrate deformation induced by vacuum cup arrays. Top images show optical views of suction cups with different radii (R). Scale bar 4 mm. Bottom images show the corresponding GelSight deformation profiles generated by each cup. Scale bar: 3 mm.

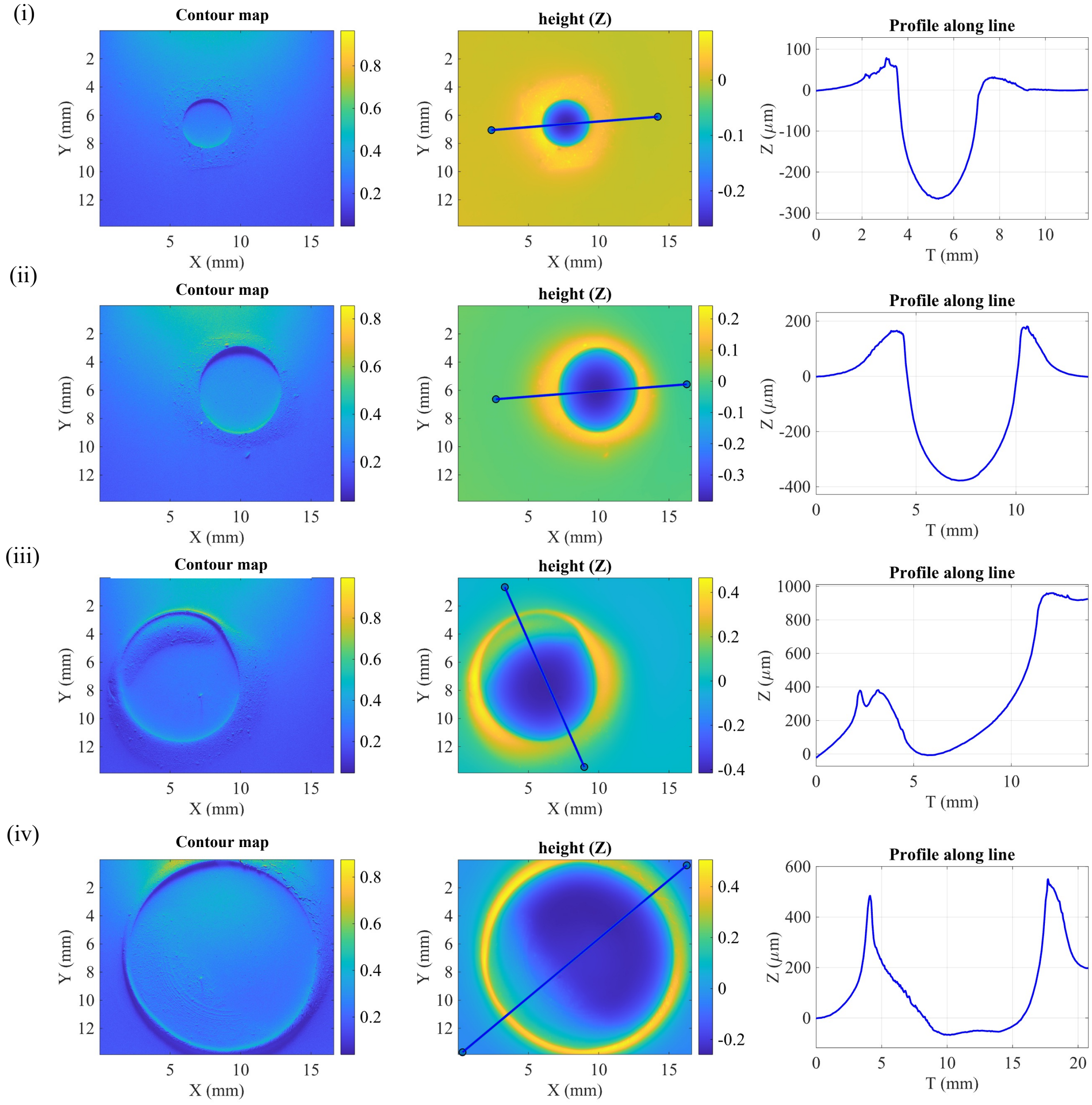

Figure S15: Effects of cup width on compliant surface deformation. Gelsight measurement and analysis for cups with fixed $R = 2mm$, t= $0.3R$ and varied a: (i) $a = 0R$, (ii) $a = 0.5R$, (iii) $a = 1R$, (iv) $a = 2R$.

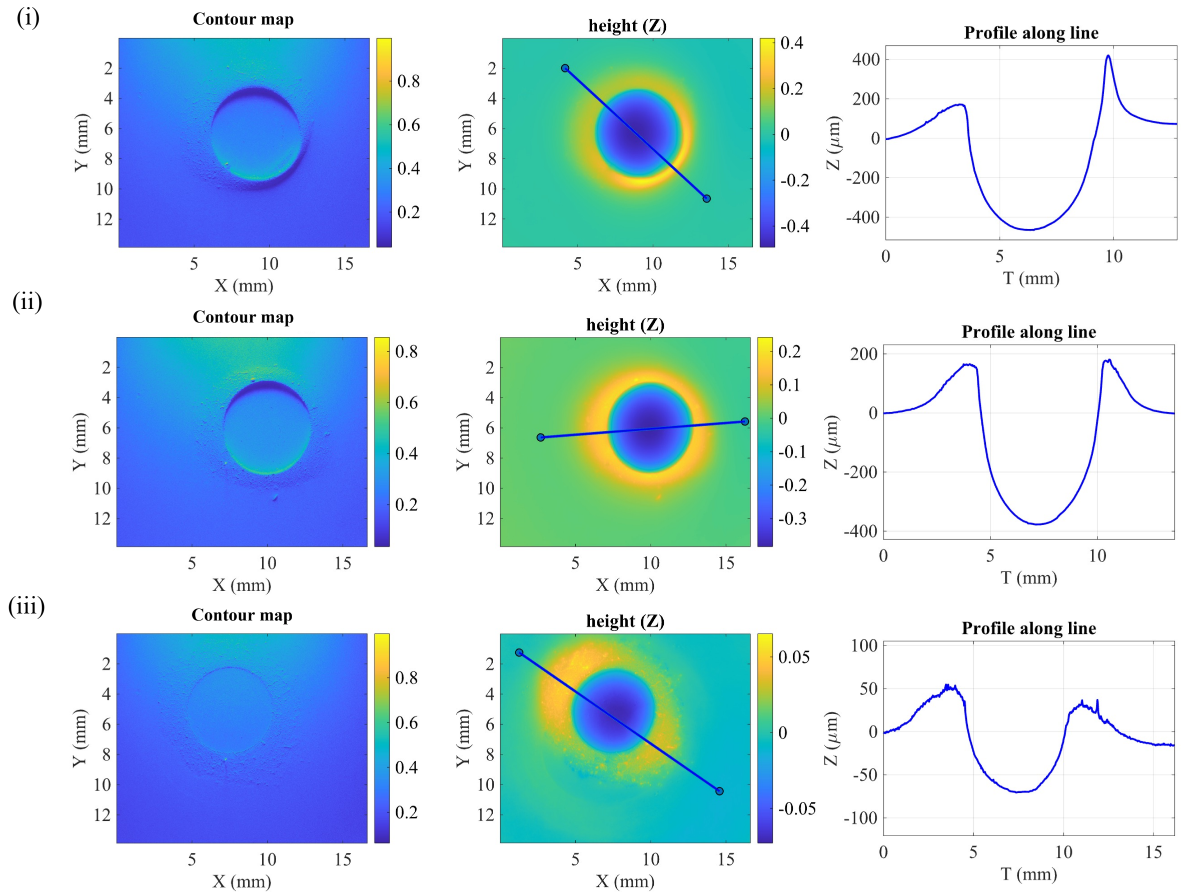

Figure S16: Effects of cup thickness on compliant surface deformation. Gelsight measurement and analysis for cups with fixed $R = 2mm$, $a = 0.5R$ and varied t: (i) $t = 0.2R$, (ii) $t = 0.3R$, (iii) $t = 0.4R$.

6 Work of adhesion measurement for interfacial materials

(i)

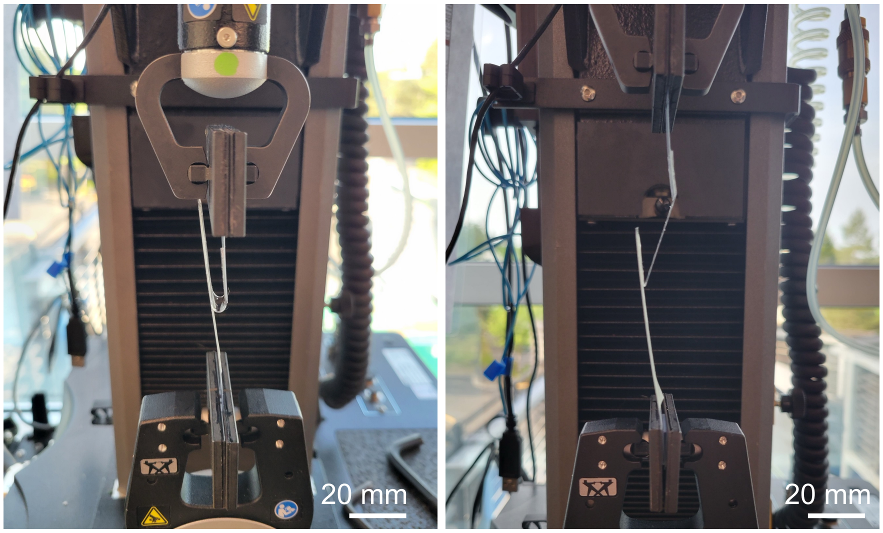

(ii)

Interfacial Materials	Modulus (kPa)	Work of Adhesion (J/m^2)
PDMS 10:1	997 ± 30	0.895 ± 0.049
PDMS 20:1	475 ± 23	0.839 ± 0.091
Ecoflex 00-20	41 ± 1	0.791 ± 0.020
Ecoflex Gel	20 ± 2	1.479 ± 0.028

Figure S17: (i)Experimental set-up for the peel test to characterize the work of adhesion between interface materials and the artificial skin substrate. (ii) Summary of measured moduli and adhesion energies for each material. Values represent the mean and standard deviation from three independent samples per condition.

7 Real contact measurement

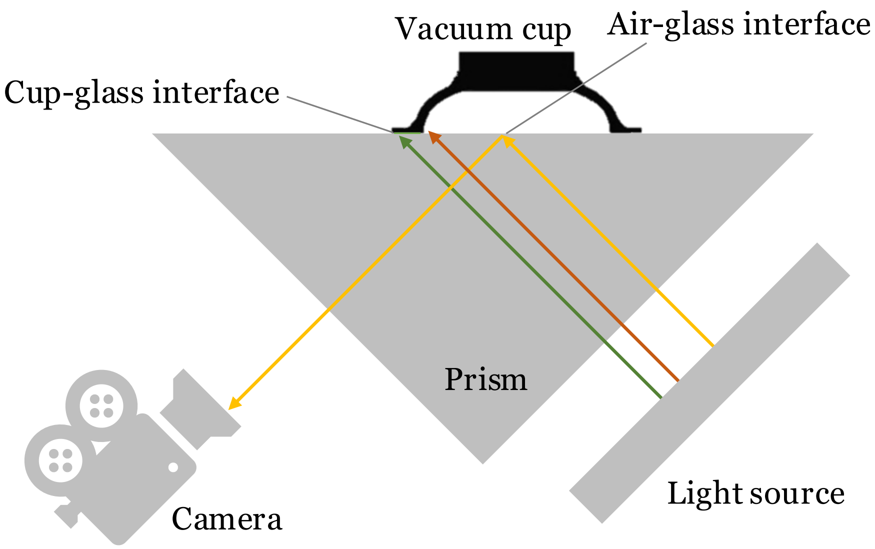

Figure S18: Real contact area measurement setup.

The setup for contact area measurement is based on total internal reflection of light when traveling from a glass with high refractive index ($n_i = 1.51509$ for N-BK7 at 632.8nm) to air with low refractive index ($n_r = 1.00028$ at 632.8nm) at an angle that is higher than the critical angle given by $\sin\theta_{air} = n_r/n_i \;\rightarrow\; \theta_{air} = 41.3°$. As shown in the schematic of the measurement setup in Fig. S18, the light from the white collimated light source hits the hypotenuse of the right-angle prism at 45°, larger than the critical angle $\theta_{air} = 41.3°$, and therefore reflects and arrives at the camera. However, when a surface such as vacuum cup contacts the glass surface, at the interface between glass and vacuum cup the light does not reflect and gets absorbed by the vacuum cup, and therefore the camera sees these contact areas as darker regions, having the same color as the vacuum cup. The setup is made using a right-angle prism with N-BK7 glass (50 mm, Uncoated, N-BK7 Right Angle Prism, Stock #32-535, Edmund Optics), collimated white backlight (2"×2" White Metaphase Technologies Collimated LED Backlight, #37-082, Edmund Optics), off-the-shelf camera (MOKOSE 4K@30fps USB Camera with 6-12 mm Varifocal Manual Lens Webcam UVC), and a custom 3D-printed fixture.

8 ECG/EMG experiment and data processing

The conductive footing layer is intentionally made significantly larger than the size of the vacuum cup array for electrical connection as shown in Fig. S19. A piece of copper tape is taped on a portion of the dangling footing layer to make it locally stiffer and increase the area of electrical connection. Then the connectors from the ECG/EMG wires are clamped onto the copper tape as shown in Fig. 4 of the main paper. ECG and EMG biosignals are recorded from the Analog Front End (AFE) of a commercial wireless surface EMG system (Ultium EMG, Noraxon USA Inc., Scottsdale, AZ) with a built-in 24 bit ADC that has a sensitivity of 300 nV resolution. A sampling rate of 4000 Hz is programmed during the entire data collection. For

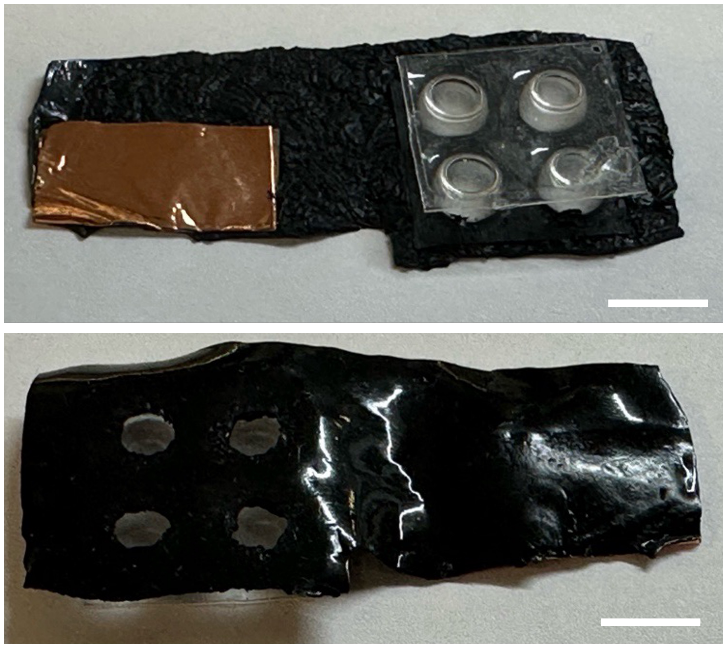

Figure S19: 2×2 suction cup array with $R = 2mm$, $a = 0.5R$. and $t = 0.3R$, coated with a conductive footing layer for ECG/EMG measurement. Scale bar 10mm

EMG measurement, an EMG sensor is attached to the body as a ground reference, a pair of soft electrodes with vacuum cups are attached to the forearm/wrist area to form a bipolar sensing. Then the user performs various muscle activities (such as maximum voluntary contraction, index-thumb finger pinch and release) while EMG signals are recorded and post-processed. The EMG envelope is post-processed via RMS with a 500 ms moving window and a bandpass filter of 10 to 1000 Hz. ECG measurement setup follows the dual-wrist ECG configuration, where the two sensing electrodes are placed on separate hands on each side of the heart so that cardiac action potential can be measured. Specifically, the ground reference and one sensing electrode are placed on the left wrist and the other sensing electrode is placed on the right wrist. The ECG raw signal is post-processed with a bandpass filter of 0.05 to 300 Hz.

9 VR system set-up

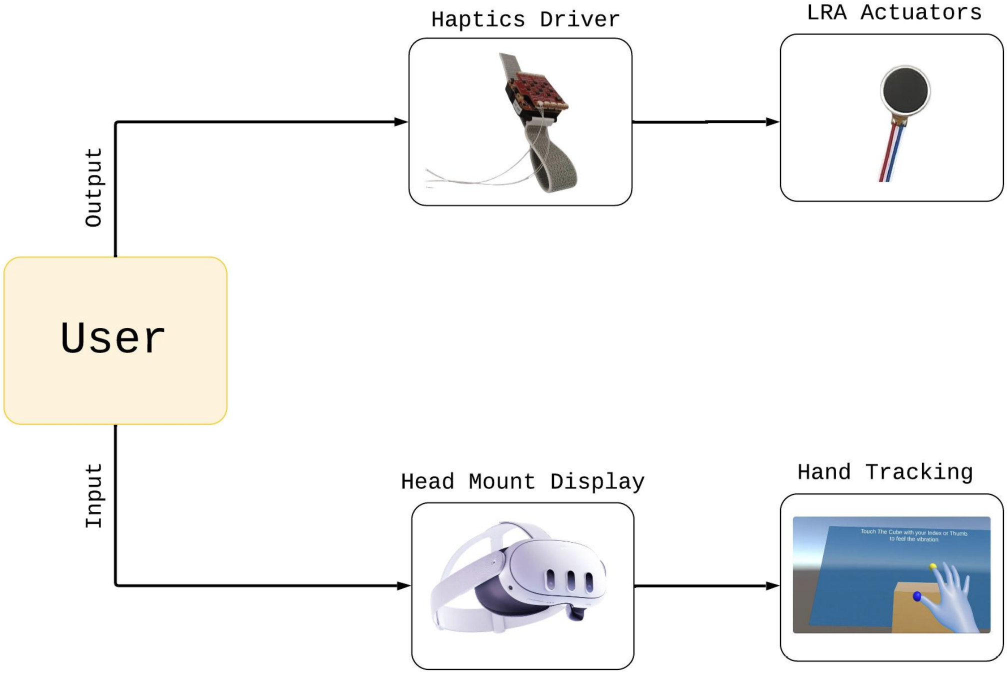

Figure S20: VR setup for tactile feedback. User wears head-mounted display with hand tracking. Touching the floating cube triggers haptic feedback via LRAs.

As an example of a use case, we develop a simple virtual reality environment that enables users to interact with virtual objects and receive tactile haptic feedback produced by actuators that are mounted on the user's hand via our suction cups. The environment includes a floating cube that can be interacted with using the thumb and index fingers. Users wear a wristband that contains the haptic driver connected to two Linear Resonant Actuators (LRAs), which are attached to the index finger and thumb via suction cups. When the user touches the floating cube with their thumb or index finger in the virtual environment, the corresponding actuator is activated through the driver, providing a sense of tactile response.

10 Movies

1. Double-sided vacuum cup arrays for weight lifting by optimizing cup geometries for hard and soft substrates.
   Movie S1.mp4
2. Vacuum cup integration with an inertial measurement unit (IMU) for motion sensing on human fingernails.
   Movie S2.mp4
3. Vacuum cup-mounted linear resonant actuators (LRA) delivering localized haptic feedback during virtual reality interaction.
   Movie S3.mp4
4. Enhanced contact of a wearable wristband using vacuum cups.
   Movie S4.mp4
5. Electromyography (EMG) signal acquisition using suction cups with conductive footing layers.
   Movie S5.mp4
